# The effect of long-term homocysteine-lowering on carotid intima-media thickness and flow-mediated vasodilation in stroke patients: a randomized controlled trial and meta-analysis

**DOI:** 10.1186/1471-2261-8-24

**Published:** 2008-09-20

**Authors:** Kathleen Potter, Graeme J Hankey, Daniel J Green, John Eikelboom, Konrad Jamrozik, Leonard F Arnolda

**Affiliations:** 1 School of Medicine and Pharmacology, University of Western Australia, Perth, Australia; 2 Department of Neurology, Royal Perth Hospital, Perth, Australia; 3 School of Sport Science, Exercise and Health, University of Western Australia, Perth, Australia; 4 Research Institute for Sport and Exercise Science, Liverpool John Moores University, Liverpool, UK; 5 Department of Medicine, McMaster University, Hamilton, Canada; 6 School of Population Health and Clinical Practice, University of Adelaide, Adelaide, Australia; 7 Department of Cardiology, Royal Perth Hospital, Perth, Australia

## Abstract

**Background:**

Experimental and epidemiological evidence suggests that homocysteine (tHcy) may be a causal risk factor for atherosclerosis. B-vitamin supplements reduce tHcy and improve endothelial function in short term trials, but the long-term effects of the treatment on vascular structure and function are unknown.

**Methods:**

We conducted a sub-study of VITATOPS, a randomised, double-blind, placebo-controlled intervention trial designed to test the efficacy of long term B-vitamin supplementation (folic acid 2 mg, vitamin B_6 _25 mg and vitamin B_12 _0.5 mg) in the prevention of vascular events in patients with a history of stroke. We measured carotid intima-medial thickness (CIMT) and flow-mediated dilation (FMD) at least two years after randomisation in 162 VITATOPS participants. We also conducted a systematic review and meta-analysis of studies designed to test the effect of B-vitamin treatment on CIMT and FMD.

**Results:**

After a mean treatment period of 3.9 ± 0.9 years, the vitamin-treated group had a significantly lower mean plasma homocysteine concentration than the placebo-treated group (7.9 μmol/L, 95% CI 7.5 to 8.4 versus 11.8 μmol/L, 95% CI 10.9 to 12.8, p < 0.001). Post-treatment CIMT (0.84 ± 0.17 mm vitamins versus 0.83 ± 0.18 mm placebo, p = 0.74) and FMD (median of 4.0%, IQR 0.9 to 7.2 vitamins versus 3.0%, IQR 0.6 to 6.6 placebo, p = 0.48) did not differ significantly between groups. A meta-analysis of published randomised data, including those from the current study, suggested that B-vitamin supplements should reduce CIMT (-0.10 mm, 95% CI -0.20 to -0.01 mm) and increase FMD (1.4%, 95% CI 0.7 to 2.1%). However, the improvement in endothelial function associated with homocysteine-lowering treatment was significant in short-term studies but not in longer trials.

**Conclusion:**

Although short-term treatment with B-vitamins is associated with increased FMD, long-term homocysteine-lowering did not significantly improve FMD or CIMT in people with a history of stroke.

**Trial Registration:**

Clinical Trial Registration URL:

Trial Registration number: 12605000005651

## Background

An elevated plasma homocysteine concentration (tHcy) is associated an increased risk of myocardial infarction and stroke [1-4]. It remains unclear, however, whether tHcy is a modifiable causal risk factor for vascular disease or simply a marker of risk burden. If homocysteine causes vascular damage, lowering tHcy ought to slow the progression of atherosclerosis and prevent atherothrombotic events. Studies that have reduced plasma homocysteine concentrations with B-vitamin supplements have produced conflicting results. Several short-term intervention studies show that lowering tHcy improves the surrogate vascular outcomes of carotid intima-media thickness (CIMT) and flow-mediated dilation (FMD) [5-10]. By contrast, B-vitamin treatment has not reduced vascular mortality or morbidity, with the possible exception of ischemic stroke, in the large randomised clinical trials published to date [11,12].

The aim of this study was to determine whether long-term homocysteine-lowering treatment with folic acid, vitamin B_6 _and vitamin B_12 _would reduce CIMT and increase FMD in subjects with a history of stroke. We also carried out a systematic review and meta-analysis to obtain an estimate of the effect of B-vitamin treatment on these vascular measurements based on the totality of published randomised evidence.

## Methods

The study was conducted in accordance with the Declaration of Helsinki. The Royal Perth Hospital Ethics Committee approved the study protocol and each subject gave written informed consent prior to taking part.

### Participants

Study subjects were recruited from participants in the Vitamins TO Prevent Stroke (VITATOPS) trial. VITATOPS is a large, international, randomised, double-blind, placebo-controlled clinical trial designed primarily to examine the efficacy and safety of B-group vitamins in the prevention of stroke, myocardial infarction or death from any vascular cause among patients with previous stroke or transient ischemic attack (TIA). The inclusion and exclusion criteria for the VITATOPS trial have been reported previously [13]. VITATOPS has enrolled a total of 976 people in Australia and almost 8000 people worldwide. The trial is expected to report its findings in 2010.

### Design

The current study was a cross-sectional single-centre sub-study of the VITATOPS trial. All VITATOPS participants enrolled in Perth, Australia between 1998 and 2003 were eligible for inclusion (n = 532). Subjects who had died, withdrawn from VITATOPS, were severely handicapped or had moved away from Perth were excluded (n = 189). Recruitment letters were sent to all subjects suitable for inclusion (n = 343). Subjects who responded positively to the letter were contacted by telephone and enrolled in this study (n = 173). The ethics approval for the project stipulated that we should not contact subjects who did not respond to the invitation letter, so non-responders were not followed up.

### Intervention

Participants in the VITATOPS trial were randomly assigned at baseline to a single daily tablet containing folic acid 2 mg, vitamin B_6 _25 mg, and vitamin B_12 _500 μg or a matching placebo. A central 24-hour telephone service and an interactive website used random permuted blocks stratified by hospital to allocate a treatment pack number.

### Objectives

We aimed to determine whether long-term (greater than two years) homocysteine-lowering treatment with B-vitamin supplements would reduce CIMT and increase FMD compared with placebo in subjects with a history of stroke or TIA.

### Outcomes

CIMT and FMD were the outcome measures chosen for this sub-study. The vascular outcomes were not measured at baseline and were assessed at a single time point at least two years after randomisation. All participants attended a morning appointment after an overnight fast. They were also asked to withhold their morning medications, including the VITATOPS tablet, and to refrain from smoking and drinking alcohol or caffeine-containing beverages for at least six hours prior to the study appointment. Age, gender, details of the primary event, medications, cardiovascular risk factors and smoking history were recorded and blood pressure, height, weight, waist and hip girth and FMD and CIMT were measured. A fasting blood sample was collected to assess tHcy, red blood cell folate, serum B_6 _and B_12_, total cholesterol, high- and low-density lipoprotein (HDL and LDL) cholesterol, triglycerides, creatinine and glucose.

CIMT and FMD were measured using techniques described previously [14,15]. Briefly, the subject lay supine on a flat examination couch in a quiet laboratory and B-mode ultrasound images were recorded using a 10 MHz multi-frequency linear array probe attached to a high-resolution ultrasound (Acuson Aspen, Mountain View, California). Digital images of the right and left common carotid arteries immediately proximal to the bifurcation were obtained from posterior, lateral and anterior angles. Mean CIMT was measured off-line using edge-tracking software. The final CIMT values for each subject were calculated as the mean of six measurements. A previous study by our group indicates that the intra-observer coefficient of variation for CIMT measured using this method is 2.4% [14].

FMD was measured in the left arm except in subjects with a left hemiplegia (n = 18). B-mode images of the brachial artery were obtained and recorded continuously for one minute to assess baseline arterial diameter. A rapid inflation/deflation pneumatic cuff placed around the forearm was inflated to 250 mmHg for five minutes. Post cuff-deflation images were recorded continuously for two minutes to capture peak arterial dilation. When fifteen minutes had elapsed, a second scan was recorded to re-establish baseline diameter. 400 μg of sub-lingual glyeryl trinitrite (GTN) was administered to the subject and images were recorded for eight minutes. FMD and GTN-mediated dilation were measured off-line using edge-detection software designed to minimise observer biases. A single observer blinded to treatment status performed all measurements. The mean intra-observer CV's for FMD and GTN-mediated dilation measured by this technique are 6.7% and 3.9% respectively [15].

### Sample Size

We aimed to recruit sufficient subjects from the Perth VITATOPS cohort to provide 80% power (α = 0.05) to detect differences of 0.1 mm in mean CIMT and 2% in FMD between the vitamin and placebo groups. Post-hoc calculations for a one-sided test showed that the study had 80% power (α = 0.05) to detect a reduction in mean CIMT of 0.07 mm and an absolute increase in FMD of 1.6%.

### Statistical analysis

As the FMD data had a non-normal distribution and multiple zero values, we used a two-sample rank sum test (Mann-Whitney) to compare the median FMD in the treatment and placebo groups. Two-sample t-tests were used to compare all normally distributed variables and results are reported as mean ± standard deviation. Variables with log-normal distributions were log-transformed and results for these variables are reported as geometric mean (95% confidence interval). Proportions were compared using a normal approximation to the binomial. Effect size was calculated as the standardised difference between treatment group means (Cohen's d) using Hedges' correction for bias. Minitab (Version 14.2, Minitab Inc, USA) and SPSS (Version 15.0, SPSS Inc, USA) were used for statistical analyses. A single observer blinded to treatment group conducted the statistical analysis.

### Systematic review and meta-analysis

**Figure 1:**
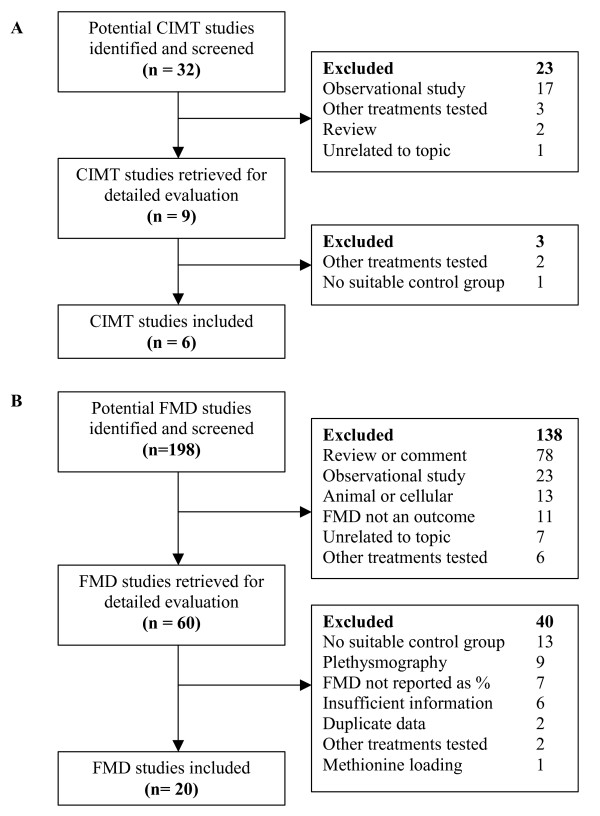
**Flow chart showing the meta-analysis paper selection process and exclusion reasons**. A CIMT paper-selection flow chart. B FMD paper-selection flow chart.

We conducted a systematic review and meta-analysis of randomised controlled trials reporting the effect of homocysteine-lowering treatment with B-vitamin supplements on FMD and CIMT. Papers were identified in the Medline (1950 to January 2008) and EMBASE (1988 to January 2008) databases and with a manual search of citations in the relevant retrieved papers. The search strategy was (*carotid intima media *or *intima medial thickness *or *IMT*) AND (*folate *or *folic acid *or *folic*) for CIMT papers and (*flow-mediated dilation *or *endothelial function *or *FMD*) AND (*folate *or *folic acid *or *folic*) for the FMD papers. Figure 1 shows the paper selection process for both analyses. We included all randomised, placebo-controlled, double-blind studies and open-label trials where observers assessing the outcomes were blinded to treatment allocation. We excluded studies that reported FMD as an absolute change in brachial artery diameter because accurate standard deviations could not be calculated for the percent change. The number of subjects, the post-treatment mean and the standard deviation in CIMT (mm) and FMD (%) in the vitamin and placebo groups were entered into the meta-analysis software (Review Manager (RevMan) software, version 4.2.10, Copenhagen, The Cochrane Collaboration). Pooled estimates were calculated from weighted mean differences using a random effects model. Sub-group analyses by treatment period and subject type were conducted for the FMD studies. We performed sensitivity analyses by deleting studies one at a time, creating funnel plots, and comparing the results of a fixed effect model with those from a random effects model.

## Results

### Subject recruitment and participant flow

**Figure 2:**
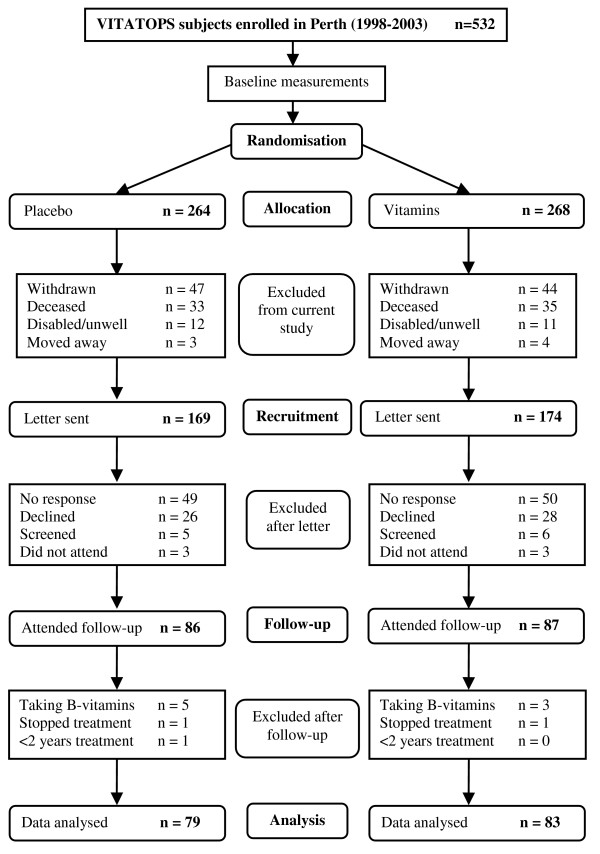
Flow chart showing the recruitment process for this study.

Subjects recruited for this sub-study initiated treatment between November 1998 and December 2003. Follow-up data was collected between August 2004 and July 2006 after a minimum of two years of continuous treatment. Participant flow is shown in Figure 2. The proportion of VITATOPS subjects excluded due to withdrawal, death, disability or geographical inaccessibility was similar in the treatment and placebo groups. Approximately half of the subjects eligible for inclusion in this sub-study agreed to participate and had follow-up data collected. A greater proportion of the participants were male and almost half as many participants as non-participants had had a haemorrhagic stroke. No other significant differences between participants and non-participants were detected in the clinical characteristics recorded at enrolment (Table 1).

**Table 1:** Demographic and clinical characteristics of subjects eligible for inclusion

	**Participants **(n = 173)	**Non-participants **(n = 170)	**p-value**
Age at event (years)	65 ± 11	64 ± 14	0.58
Sex (male)	126 (73)	96 (56)	< 0.01
**Stroke type**			
Ischemic	124 (72)	117 (69)	0.33
TIA	34 (20)	28 (16)	
Haemorrhagic	13 (7)	22 (13)	
Unknown	2 (1)	3 (2)	
**Medications**			
Antiplatelet agent	140 (81)	131 (77)	0.40
Warfarin	28 (16)	30 (18)	0.76
Statin	57 (33)	51 (30)	0.54
Antihypertensive agent	97 (56)	83 (49)	0.20
**Risk factors**			
Hypertension*	120 (69)	105 (62)	0.14
Hypercholesterolemia^†^	61 (35)	50 (30)	0.31
Diabetes^‡^	29 (17)	32 (19)	0.71
Current smoker	33 (19)	31 (18)	0.84
**Clinical characteristics**			
Systolic BP (mmHg)	133 ± 18	134 ± 18	0.75
Diastolic BP (mmHg)	78 ± 10	78 ± 10	0.78
GFR (ml/min/1.73 m^2^)^§^	87 ± 21	90 ± 27	0.35

Values are mean ± standard deviation or number (%)

P-values for continuous variables are from a two-sample t-test, from a normal approximation to the binomial for proportions and from a chi-square test for categories.

TIA indicates transient ischemic attack of eye or brain; BMI, body mass index; BP, blood pressure, GFR, glomerular filtration rate.

*History of previous or current hypertension

^†^Treated for hypercholesterolaemia

^‡ ^Includes type I, type II and impaired fasting glycaemia

^§^Estimated using the Modification of Diet in Renal Disease Study equation [32]

### Numbers analysed

173 subjects had follow-up data collected. We conducted an on-treatment analysis and thus excluded data from eleven subjects, seven from the placebo group and four from the vitamin group. Eight subjects were taking open-label supplements containing folic acid or B-vitamins, two had stopped taking the VITATOPS tablets and one had been taking the VITATOPS medication for less than two years. Results from four additional subjects were excluded from the FMD analysis, two from the placebo group (poor scan quality and cigarette smoking less than six hours prior to the study session) and two from the vitamin group (food intake and cigarette smoking less than six hours prior to the study session).

### Baseline and follow-up data

The demographic and clinical characteristics of each group at baseline and follow-up are presented in Table 2. There were no significant differences in these variables between the groups at baseline or follow-up. The systolic blood pressure appeared to be lower in the vitamin group at follow-up, but the difference was not significant when corrected for baseline values (p = 0.19). Blood results are presented in Table 3. Mean tHcy was lower at baseline in the subjects randomised to vitamins and had dropped by a further 23% at follow-up. As expected, the vitamin group had significantly higher red blood cell folate, serum B_6 _and serum B_12 _levels at follow-up than the placebo group.

**Table 2:** Subject characteristics at baseline and follow-up

	**Baseline**		**Follow-up**	
	**Placebo **(n = 79)	**Vitamins **(n = 83)	**Placebo **(n = 79)	**Vitamins **(n = 83)
Age (years)	65 ± 11	64 ± 12	69 ± 11	68 ± 12
Sex (male)	56 (71)	61 (73)	56 (71)	61 (73)
Treatment period (years)	-	-	3.8 ± 0.9	4.0 ± 0.9
**Stroke type**				
Ischemic	54 (69)	62 (75)	-	-
TIA	19 (24)	13 (16)	-	-
Haemorrhagic	4 (5)	8 (10)	-	-
Unknown	2 (3)	0 (0)	-	-
**Medications**				
Antiplatelet agent	68 (86)	63 (76)	60 (76)	59 (71)
Warfarin	11 (14)	16 (19)	13 (17)	15 (18)
Statin	24 (32)	30 (36)	59 (75)	56 (68)
Antihypertensive agent	47 (60)	45 (54)	69 (87)	71 (86)
**Risk factors**				
Hypertension*	57 (72)	63 (75)	69 (87)	71 (86)
Hypercholesterolemia^†^	25 (32)	29 (35)	59 (75)	56 (68)
Diabetes^‡^	14 (18)	15 (18)	22 (28)	19 (23)
Current smoker	15 (19)	18 (22)	7 (9)	7 (8)
Fruit (pieces/day)	-	-	1.7 ± 1.3	1.6 ± 1.1
Vegetables (serves/day)	-	-	3.5 ± 1.1	3.5 ± 1.2
**Clinical characteristics**				
BMI (kg/m^2^)	-	-	29.3 ± 4.9	29.1 ± 4.8
Waist circumference (cm)	-	-	103 ± 12	103 ± 13
Systolic BP (mmHg)	135 ± 15	132 ± 16	145 ± 19	140 ± 16
Diastolic BP (mmHg)	78 ± 9	78 ± 10	79 ± 11	78 ± 10
GFR (ml/min/1.73 m^2^)^§^	84 ± 19	88 ± 21	79 ± 18	77 ± 24

Values are mean ± standard deviation or number (%)

TIA indicates transient ischemic attack of eye or brain; BMI, body mass index; BP, blood pressure, GFR, glomerular filtration rate.

*History of previous or current hypertension

^†^Treated for hypercholesterolaemia

^‡ ^Includes type I, type II and impaired fasting glycaemia

^§^Estimated using the Modification of Diet in Renal Disease Study equation [50]

**Table 3:** Blood results at baseline and follow-up

	**Baseline**		**Post-treatment**		**Adjusted mean treatment difference**	**p-value**
	**Placebo **(n = 79)	**Vitamins **(n = 83)	**Placebo **(n = 79)	**Vitamins **(n = 83)		
**tHcy **μmol/L	11.7 (10.9, 12.6)	10.3 (9.6, 11.1)	11.8 (10.9, 12.8)	7.9 (7.5, 8.4)	-3.7 ± 0.8	< 0.001
**Serum B**_6 _nmol/L	40 (36, 45)	34 (31, 38)	34 30, 38)	118 (116, 121)	79 ± 3	< 0.001
**Serum B**_12 _pmol/L	297 (266, 331)	317 (285, 351)	274 (248, 302)	638 (600, 680)	328 ± 25	< 0.001
**RBC folate **nmol/L	805 (732, 885)	884 (796, 982)	944 (839, 1061)	2534 (2401, 2673)	1475 ± 103	< 0.001
**Cholesterol **mmol/L	4.7 (4.5, 4.9)	4.8 (4.6, 5.0)	4.3 (4.2, 4.5)	4.4 (4.2, 4.6)	-0.1 ± 0.2	0.63
**Triglycerides **mmol/L	1.4 (1.2, 1.5)	1.5 (1.3, 1.6)	1.3 (1.1, 1.4)	1.3 (1.1, 1.4)	-0.1 ± 0.1	0.53
LDL* mmol/L	-	-	2.3 (2.1, 2.4)	2.3 (2.1, 2.4)	-	-
**HDL **mmol/L	-	-	1.3 (1.2, 1.4)	1.3 (1.2, 1.3)	-	-
**Creatinine **μmol/L	80 (76, 83)	79 (74, 84)	84 (81, 88)	88 (83, 90)	3.1 ± 3.0	0.30
**Cystatin C **mg/L	1.02 (0.97, 1.07)	1.05 (1.00, 1.11)	1.04 (0.99, 1.09)	1.08 (1.02, 1.15)	0.01 ± 0.03	0.69
**GGT** mmol/L	-	-	26 (22, 30)	30 (27, 34)	-	-
**Glucose **mmol/L	5.4 (5.3, 5.6)	5.5 (5.3, 5.7)	5.1 (4.9, 5.2)	5.2 (5.0, 5.4)	0.1 ± 0.2	0.59
**HbA1c **%	-	-	5.8 (5.6, 6.0)	5.9 (5.7, 6.1)	-	-

RBC indicates red blood cell; LDL, low density lipoprotein; HDL, high density lipoprotein; GGT, gamma glutamyl transpeptidase; HBA1c, glycated haemoglobin.

Baseline and follow-up values are presented as geometric mean (95% confidence interval)

Adjusted mean treatment differences are presented as mean ± standard error

P-values for the adjusted mean treatment differences are from a univariate general linear model.

* Calculated using the Friedewald equation

### Vascular outcomes and estimated effect size

The vascular outcome measurements are presented in Table 4. The mean CIMT in the vitamin group was not significantly different from the mean CIMT in the placebo group at follow-up. The mean difference between groups was 0.01 mm (95% CI -0.04 mm, 0.06 mm). The estimated effect size was 0.06 (95% CI -0.25, 0.37). The median FMD in the vitamin group was not significantly different from the median FMD in the placebo group at follow-up. The mean difference between the groups was 0.5% (95% CI -0.73%, 1.73%). The effect size was 0.12 (95% CI -0.19, 0.44). We did not find any significant differences in the effect of B-vitamins on CIMT or FMD among the etiological subtypes of stroke. Results from an intention-to-treat analysis were not substantially different from the on-treatment analysis reported here.

**Table 4:** Vascular outcome measurements

	**Placebo (n = 79)**	**Vitamins (n = 83)**	**p**
**Vascular Structure **			
Carotid intima-medial thickness (mm)	0.83 ± 0.18	0.84 ± 0.17	0.74
Carotid lumen diameter (mm)	6.35 ± 0.72	6.49 ± 0.94	0.31
Brachial artery diameter (mm)	3.66 ± 0.67	3.84 ± 0.82	0.12
**Vascular Function **			
Flow-mediated dilation (%)	3.0 (0.6 – 6.6)	4.0 (0.9 – 7.2)	0.48
GTN-mediated dilation (%)	20.2 ± 7.4	20.2 ± 8.1	0.98

GTN indicates glyeryl trinitrate.

Values are mean ± standard deviation or median (inter-quartile range)

P-values are from a two-sample t-test or a two-sample rank test (Mann-Whitney)

### Systematic review and meta-analysis

**Figure 3:**
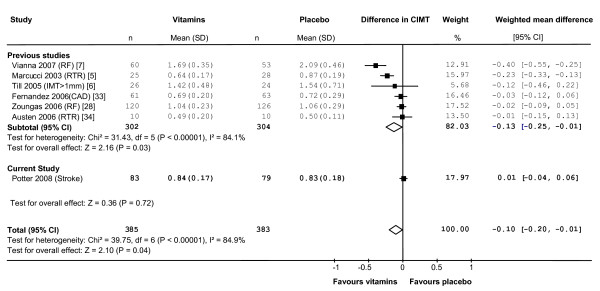
**Change in CIMT associated with B-vitamin treatment in all randomised trials published to date**. Squares indicate the mean difference in CIMT between the vitamin-treated and placebo-treated groups: the size of the square is proportional to the number of subjects in the study and the horizontal line indicates the 95% confidence interval. Diamonds represent the 95% confidence intervals for the sub-total and total differences. Studies are ordered by effect size. WMD, weighted mean difference; CI, confidence interval; RF indicates subjects with renal failure as defined by authors; RTR, renal transplant recipients; CAD, coronary artery disease [5-7,28,33,34]

**Figure 4:**
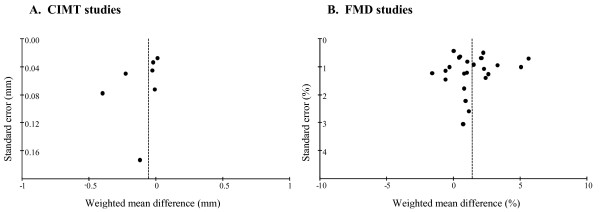
**Funnel plots for CIMT and FMD meta-analyses**. A Standard error (mm) plotted against weighted mean difference (mm) for all CIMT studies included in meta-analysis. B. Standard error (%) plotted against weighted mean difference (%) for all FMD studies included in meta-analysis.

We identified six randomised placebo-controlled studies that measured the effect of B-vitamin treatment on CIMT. The pooled estimate from these studies was a significant reduction in CIMT of 0.13 mm (95%CI -0.25, -0.01 mm, n = 606, p = 0.03, I^2 ^= 84.1%) in vitamin-treated subjects compared with placebo (Figure 3). The inclusion of our data reduced the estimated effect to -0.10 mm and narrowed the confidence interval to -0.20 to -0.01 mm (n = 768, p = 0.04, I^2 ^= 84.9%). Using a fixed-effect versus a random-effects model did not substantially alter the pooled estimate. However, removing the Vianna (2007) data from the analysis reduced the pooled estimate to non-significance (-0.06 mm, 95% CI -0.13, 0.02) [7]. A funnel plot of effect size versus study precision was asymmetrical with a relative dearth of moderately precise negative studies, suggesting the presence of a positive publication bias (Figure 4A).

**Figure 5:**
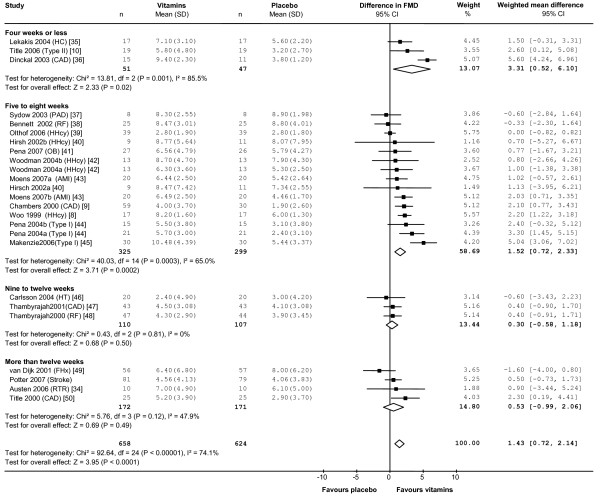
**Change in FMD associated with B-vitamin treatment in previous randomised controlled trials**. Squares indicate the mean difference in FMD between the vitamin and placebo groups: the size of the square is proportional to the number of subjects in the study and the horizontal line indicates the 95% confidence interval. Diamonds represent the 95% confidence intervals for the sub-total and total differences. Studies are ordered by effect size. WMD indicates weighted mean difference; CI, confidence interval; HC indicates subjects with hypercholesterolaemia as defined by study authors; Type II, type II diabetes mellitus; CAD, coronary artery disease; PAD, peripheral artery disease; RF, renal failure; HHcy, hyperhomocysteinemia; OB, obese; AMI, acute myocardial infarction; N, normal healthy subjects; Type I, type 1 diabetes mellitus; HT, hypertension; FHx, family history of cardiovascular disease; RTR, renal transplant recipients [8-10,34-50].

We identified twenty randomised controlled trials that met our inclusion criteria for measuring the effect of B-vitamins on FMD. The pooled estimate, including our data, was an absolute increase in FMD of 1.4% (95%CI 0.7, 2.1%, n = 1280, p < 0.0001) in vitamin-treated subjects compared with placebo (Figure 5). When studies were divided by treatment period, the significant improvement in FMD in short-term studies (less than eight weeks treatment) was not apparent in longer-term studies. When the studies were analysed according to subject type, treatment with B-vitamins improved FMD in subjects with diabetes (3.5%, 95% CI 2.3, 4.7%, n = 170) and in those with coronary artery disease (1.9%, 95%CI 0.4, 3.4%, n = 347), but had no significant effect on FMD in healthy subjects with elevated tHcy (1.0%, 95%CI -0.2, 2.3%, n = 204), in subjects with renal impairment (0.2%, 95% CI -0.8, 1.3%, n = 161) or in subjects with risk factors for cardiovascular disease such as a family history of vascular disease, hypercholesterolemia or hypertension (-0.1%, 95% CI -2.1, 1.9%, n = 197).

Removing individual studies did not substantially alter the estimated effect, and nor were there any differences between results obtained using the fixed-effect versus the random-effects model. Including the seven studies that reported FMD as an absolute change in brachial artery diameter by using mean standardised differences did not change the overall result or the results of the sub-group analyses. A funnel plot of effect size against study precision appeared to be symmetrical (Figure 4B).

## Discussion

We found that long-term homocysteine-lowering with folic acid, vitamin B_6 _and vitamin B_12 _did not reduce CIMT or increase FMD in individuals with a history of stroke. When we included our results with data from similar randomised trials in a meta-analysis the estimated pooled effect was a modest improvement in both CIMT and FMD.

The 95% confidence intervals for the effect of B-vitamin supplementation on CIMT (-0.04 mm, 0.06 mm) and FMD (-0.73%, 1.73%) in our study overlap with confidence intervals from the meta-analyses of -0.25 mm to -0.01 mm and 0.73% to 2.23% (current data excluded), indicating that our results are consistent with pooled data from similar randomised studies. In a recent systematic review and meta-analysis, Lorenz et al. report that a difference of 0.1 mm in CIMT predicts a 10% to 15% reduction in the risk of future MI and a 13% to 18% reduction in the risk of future stroke [16]. To the best of our knowledge, a similar calculation for the predicative value of an absolute difference in FMD has not been reported. A recent study by Yeboah et al. reports a 9% reduction (95% CI 1%, 17%) in the risk of a future cardiovascular event (cardiovascular disease death, myocardial infarction, stroke, congestive heart failure, claudication, angioplasty or cardiac bypass graft surgery) per unit SD in FMD, an absolute difference of approximately 1.2% [17]. Our meta-analyses estimates for the effect of B-vitamin supplementation on CIMT (-0.1 mm) and FMD (+1.4%) thus appear to be consistent with a significant clinical benefit, a result that conflicts with randomised trial evidence that homocysteine-lowering treatment does not significantly reduce mortality or morbidity [12,18-22]. There are several possible explanations for this discrepancy.

One explanation is that the results of the CIMT meta-analysis are unreliable. Only a small number of studies have measured the effect of B-vitamin treatment on CIMT (n = 768 subjects, including the current study), the results show considerable heterogeneity (I^2 ^= 85%), are strongly influenced by a single study and are likely to have been influenced by publication biases. A large study (n = 819) that has not yet published detailed data has recently reported finding no difference between placebo and treatment groups in mean CIMT after three years of treatment with folic acid [23]. Adding this study to the CIMT meta-analysis would double the subject numbers and significantly reduce the estimated effect of homocysteine-lowering treatment.

By contrast with the CIMT data, the FMD meta-analysis results appear to be relatively robust with no clear evidence of a publication bias. A similar meta-analysis has also recently reported a positive effect of homocysteine-lowering treatment on FMD (1.08%, 95%CI 0.57, 1.59%, n = 732) [24]. A possible explanation for the discrepancy between the apparent improvement in FMD in the meta-analyses and the lack of improvement in clinical trial event rates is that the laboratory studies and the intervention trials have been conducted in different subject groups. Most clinical trials have recruited subjects with existing vascular disease, either coronary artery [19-21,25-27] or cerebrovascular disease [18], or with strong vascular risk factors such as diabetes [19] or renal impairment [22,28,29]. While the effect of homocysteine-lowering on FMD has also been assessed most frequently in subjects with existing disease or vascular risk factors, many studies have been conducted in healthy individuals.

It is possible that homocysteine-lowering treatment may prove more effective in individuals without established vascular disease, as a recent meta-analysis of clinical trial data suggests that the incidence of new ischemic stroke is significantly reduced by B-vitamin supplementation whilst recurrent stroke is not [11]. However, our sub-group analysis indicates that B-vitamin treatment improves FMD in people with diabetes and coronary disease but not in healthy subjects. The discrepancy between the FMD data and the clinical trial results is thus not explained by the laboratory studies having been undertaken in healthy individuals and the clinical trials in subjects with established disease.

Our sub-group analysis also indicates that the significant improvements in FMD associated with short-term folic acid treatment are not sustained in longer-term studies (Figure 5). Whether this observation reflects a true biological effect, is due to publication biases, or to systematic differences between short and long term trials with respect to the characteristics of participants or dose of folic acid dose is not clear. However, it raises the interesting question of whether long-term exposure to B-vitamins might have adverse effects on the vasculature that counteract any short-term improvement in endothelial function. The Heart Outcomes Prevention Evaluation (HOPE) 2 study found that vitamin-treated subjects were more likely to be admitted to hospital with unstable angina than those on placebo (RR 1.24, 95% CI 1.04, 1.49) [19]. This finding might be attributed to chance were it not for evidence that B-vitamin therapy increases the risk of in-stent stenosis and the need for revascularisation following angioplasty [20,21]. The Norwegian Vitamin Trial (NORVIT) in 3749 men and women with a recent myocardial infarction also found a small increase in the risk of the composite endpoint (recurrent myocardial infarction, stroke or sudden death attributed to coronary artery disease) in the vitamin group (RR 1.22, 95% CI 1.00, 1.50, p = 0.05) [27]. Excess folic acid, through its growth-promoting effects, could conceivably accelerate intimal hyperplasia and smooth muscle cell proliferation in existing vascular lesions.

A more controversial explanation for the discrepancy between the improvements in FMD and CIMT in our meta-analysis and the negative results from the clinical trials is that FMD and CIMT are not particularly reliable or sensitive indicators of cardiovascular risk. Although FMD and CIMT are widely and enthusiastically accepted as surrogates for vascular events, the evidence linking these measurements with hard clinical endpoints is relatively weak and the small changes in FMD and CIMT associated with B-vitamin treatment in our meta-analyses may actually have limited independent prognostic significance [30,31].

## Limitations

Our study has several strengths, namely its large size, long duration and randomised, placebo-controlled, double-blind design. Ours is the first study to investigate the effects of B-vitamin supplementation on FMD and CIMT in people with a history of stroke and TIA. By conducting a systematic review and meta-analysis, we were able to estimate the effect of B-vitamin treatment on FMD and CIMT based on the totality of available randomised evidence. This additional analysis puts our results in proper context and allows for the addition of more randomised data as they are published.

Our study also has limitations. We did not measure the FMD or CIMT prior to randomisation and were thus unable to adjust the post-treatment change in these measurements for baseline values. The randomisation process should have ensured that both groups had similar CIMT and FMD at baseline, making it statistically valid to estimate the effect of treatment by testing only the follow-up measurements. By chance, however, the group randomised to B-vitamins had a lower mean tHcy at baseline than the group randomised to placebo. Although the two groups were similar in all other clinical characteristics, we cannot be sure that the mean CIMT and FMD were actually the same in both groups prior to treatment. However, as tHcy is positively associated with CIMT and negatively with FMD, any baseline differences in these measurements between the groups should have increased rather than reduced the probability of finding improved FMD and CIMT in the vitamin group at follow-up.

A further limitation of the study is that, due to ethical constraints, we did not contact subjects who failed to respond to the invitation letter. We collected usable data from only 30% of the subjects originally randomised, approximately half of the subjects identified retrospectively as eligible for inclusion. Apart from a higher proportion of males and fewer haemorrhagic strokes, participants did not differ significantly from the eligible non-participants in demographic or clinical characteristics and thus appeared to be broadly representative of the larger cohort (Table 2). It is likely however, that the participants in this study comprise an intelligent, motivated and compliant sub-group of VITATOPS subjects. Most participants had well-controlled blood pressure and cholesterol levels, were on optimal medical treatment and had healthy life-styles, factors that may have reduced our ability to detect any additional vascular benefits from the homocysteine-lowering treatment.

Selecting subjects who had survived for at least two years after randomisation without severe disability may also have influenced our results. If subjects with a low FMD or high CIMT randomised to the vitamin group had improved disability-free survival compared with similar subjects randomised to placebo, they may have been over-represented in the vitamin-group at follow-up, reducing our chances of finding a between-group difference in the vascular outcomes. However, we found no difference in the number of subjects in each group excluded from the current study due to death or disability, making it unlikely that this potential source of bias substantially altered our results.

### Generalisability

It is not clear that our negative result can be extrapolated to people with a history of stroke in the general population. The majority of our subjects were on optimal medical treatment, which may have made it difficult to detect any additional benefit from lowering tHcy. In addition, the subjects were a self-selected group of trial participants and were thus a highly motivated and compliant group of individuals unlikely to be representative of the community at large.

## Conclusion

Our results indicate that long-term homocysteine-lowering treatment with B-vitamins does not significantly reduce CIMT or increase FMD in people with a history of stroke. In addition, the modest increase in FMD associated with short-term B-vitamin treatment does not appear to be translated into improved vascular structure or sustained in the longer term.

## Abbreviations

CIMT: indicates carotid intima-media thickness; FMD: flow-mediated dilation; GTN: glyeryl trinitrite; tHcy: total plasma homocysteine; VITATOPS: VITAmins TO Prevent Stroke

## Competing interests

GJH, JE and KJ are all investigators on the VITATOPS trial. The authors declare that they have no competing interests.

## Authors' contributions

GJH, JE and KJ are investigators on the VITATOPS trial. LFA, GJH, DJG and KP contributed to the design of the VITATOPS sub-study reported in this paper. KP recruited the study participants, measured FMD and CIT, collected, recorded and analysed the study data and wrote the draft manuscript. GJH, JE, DJG, KJ and LFA all critically reviewed the manuscript. All authors read and approved the final manuscript.

## Pre-publication history

The pre-publication history for this paper can be accessed here:


